# Comparative analysis of gut microbiota between common (*Macaca fascicularis fascicularis*) and Burmese (*M. f. aurea*) long-tailed macaques in different habitats

**DOI:** 10.1038/s41598-023-42220-z

**Published:** 2023-09-11

**Authors:** Raza Muhammad, Pavit Klomkliew, Prangwalai Chanchaem, Vorthon Sawaswong, Titiporn Kaikaew, Sunchai Payungporn, Suchinda Malaivijitnond

**Affiliations:** 1 Department of Biology, Faculty of Science, Chulalongkorn University, Bangkok, 10330 Thailand; 2 Center of Excellence in Systems Microbiology, Faculty of Medicine, Chulalongkorn University, Bangkok, 10330 Thailand; 3 Department of Biochemistry, Faculty of Medicine, Chulalongkorn University, Bangkok, 10330 Thailand; 4 National Primate Research Center of Thailand, Chulalongkorn University, Saraburi, 18110 Thailand

**Keywords:** Microbiology, Zoology

## Abstract

The environment has an important effect on the gut microbiota—an essential part of the host’s health—and is strongly influenced by the dietary pattern of the host as these together shape the composition and functionality of the gut microbiota in humans and other animals. This study compared the gut microbiota of *Macaca fascicularis fascicularis* and *M. f. aurea* in mangrove and island populations using 16S rRNA gene sequencing on a nanopore platform to investigate the effect of the environment and/or diet. The results revealed that the *M. f. fascicularis* populations that received anthropogenic food exhibited a higher richness and evenness of gut microbiota than the *M. f. aurea* populations in different habitats. Firmicutes and Bacteroidetes were the two most abundant bacterial phyla in the gut microbiota of both these subspecies; however, the relative abundance of these phyla was significantly higher in *M. f. aurea* than in *M. f. fascicularis*. This variation in the gut microbiota between the two subspecies in different habitats mostly resulted from the differences in their diets. Moreover, the specific adaptation of *M. f. aurea* to different environments with a different food availability had a significant effect on their microbial composition.

## Introduction

The gastrointestinal tract harbors about 10–100 trillion microorganisms, which are comprised of bacteria, viruses, fungi, and parasites, and are collectively called the gut microbiome^1,2^. Bacteria comprise more than 99% of the microorganisms in the gut, of which 1000–1150 species have been identified^3^. In the past two decades the gut microbiome has gained increasing attention as a crucial component in the host’s immune function, physiology, and even behavior^4,5^. For instance, the significance of microbial colonization in rodents through altered motor activity and anxiety-like behavior in germ-free mice compared to specific pathogen-free mice with a normal gut microbiota. Indeed, recent studies have reported that the normal gut microorganisms play a crucial role in health maintenance, including dietary metabolism, vitamin synthesis^6^, and protection against pathogens^7^. In fact, due to the important roles of the gut microbiota in the host’s physiology, it is considered as the second genome in animals^8^. Changes in the normal gut microbiota composition, called dysbiosis, have been associated with various pathologic conditions, such as inflammatory bowel diseases^9^, irritable bowel syndrome^10^, metabolic diseases, including obesity and diabetes mellitus^11^, and allergic diseases^12^.

The composition of the gut microbiota can be influenced by several factors, including host genetics and the environment (habitats and diets). Recent studies indicated that host genetics play a crucial role in shaping the gut microbiota’s composition in both humans and non-human primates (NHPs)^13–15^. For example, a study involving human twins revealed that monozygotic twins exhibited a more similar microbial composition in comparison to dizygotic twins^13^. Another study in nine folivorous wild NHP species that live in an overlapping geographical range emphasized the significant influence of diets, host gut morphology, and phylogeny on the gut microbiota composition^14^. Diet stands as one of the most influential environmental factors on the gut microbiota’s composition and is strongly linked with the habitat types that, in turn, affects the availability of various food types^15–22^.

*Macaca fascicularis* or long-tailed macaque is a NHP that is widely distributed throughout Southeast Asia and is classified into 10 subspecies based on their geographical localities and morphological characteristics^23^. Among these 10 subspecies, *M. fascicularis aurea* (*Mfa*; Burmese long-tailed macaque) has acquired attention over the past decade due to their stone-tool use behavior during foraging^24^. In southwestern Thailand, *Mfa* lives in close contact with* M. f. fascicularis* (*Mff*; common long-tailed macaque); however, *Mff* has never been reported using percussive stone tools to forage for foods in either their natural habitat^24,25^ or in captivity upon training^26^.

The genetic characteristics, based on mtDNA, Y chromosome *SRY* and *TSPY* genes, whole genome sequences, and autosomal SNPs, suggested that the two subspecies are genetically distinct^27–30^. Thus, it has been hypothesized that different genetics might be one of the factors that led to the emergence of stone-tool use in *Mfa* but not in the other subspecies^31^. With respect to the stone-tool use behavior being found only in *Mfa*, it has also been proposed that it might be due to the differences in their habitat types, which in turn could reflect the different types of food availability in their natural habitats. Note that *Mff* primarily inhabit mainland areas or its fringes, including riverbanks and mangrove forests, while *Mfa* is predominantly found on islands^23,27,32,33^. Therefore, we hypothesized that variations in the bacterial microbiomes between the two subspecies of *Macaca fascicularis* might correspond to their habitat type and dietary preferences in association with the performance of their stone-tool use behaviors.

To date, gut microbiota profiling of *Mff* has been reported, while that of *Mfa* has not yet been carried out^34–37^. Therefore, this study aimed to investigate and compare the gut microbiota in two different host groups (two phylogenetically separated subspecies of long-tailed macaques; *Mff* and *Mfa*) from two habitat types (mangrove forest and island) that acquire two different food types (anthropogenic foods and natural foods including foods acquired by stone-tool use). These findings may serve as a foundation for future microbiome research in other species of NHPs and humans.

## Results

### Food items consumed by *Mff *and *Mfa*

During the field survey and fecal specimen collection of *Mff* on July 12–21, 2022, we found that the *Mff* on Koh Ped (KPE) island and Bang Ta Boon (BTB) mangrove forest were frequently provided food (mostly as fresh fruits, such as banana, watermelon, guava, and pineapple) by tourists and local people. For KPE, it is a small island (also known as Monkey Island) situated in Chonburi province and is one of the favorite hotspots for tourists in eastern Thailand. Tourist boats and yachts regularly visited this island before the COVID-19 pandemic, with the tourists staying for a long time because of the scenic view. As a result, the tourists have access to the *Mff*-KPE and fed them. The *Mff*-BTB population inhabited a mangrove forest in Phetchaburi province, southern Thailand. Local people often visited the location and fed the monkeys.

**Figure 1:**
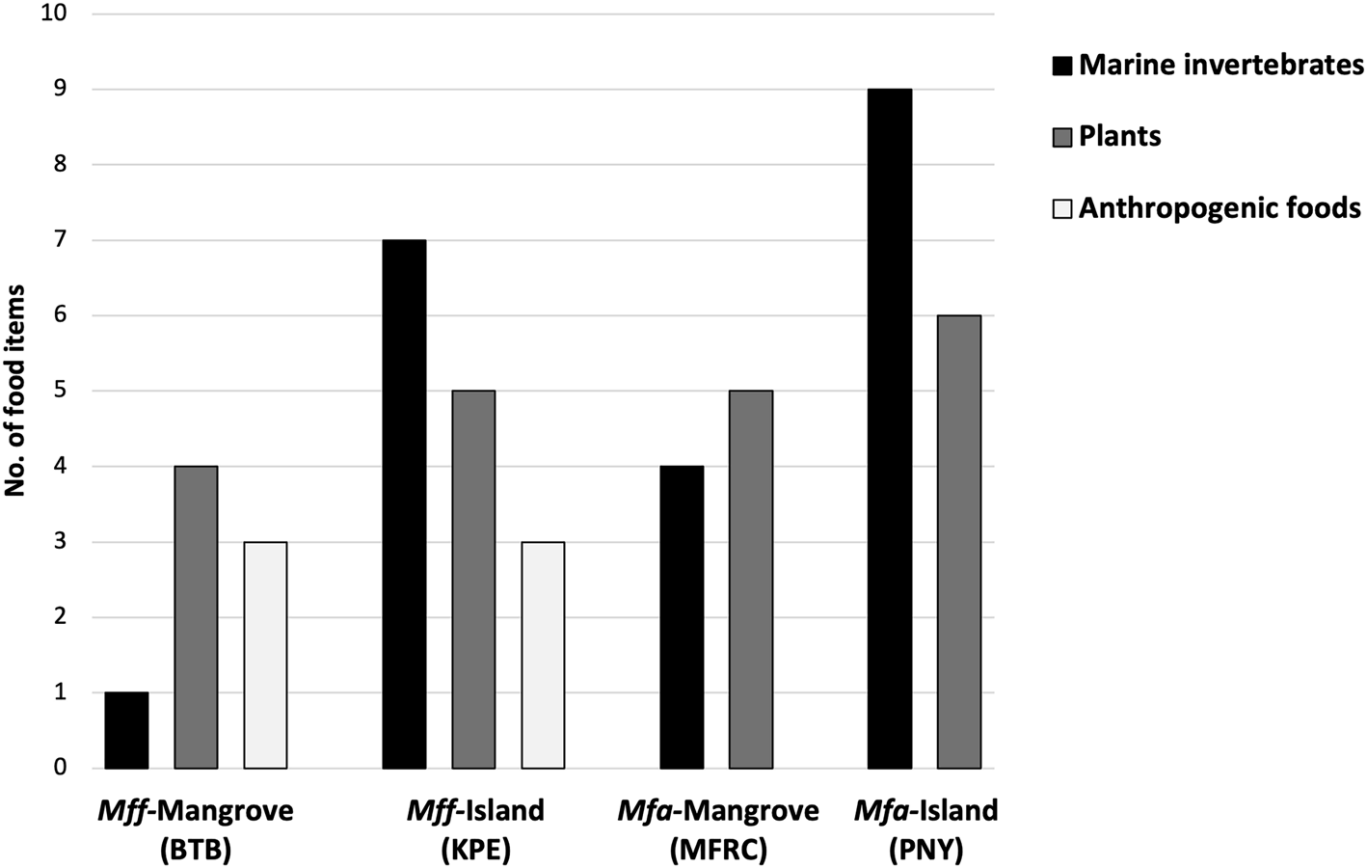
The total number of food items (per visit) consumed by *Mff* and *Mfa* living in a mangrove forest or on an island. The black, grey, and white columns indicate marine invertebrates, plants, and anthropogenic foods, respectively.

The *Mfa* were surveyed, and fecal specimens were collected between July 26 to August 06, 2022. The two *Mfa* populations lived in the Mangrove Forest Research Center (MFRC) and Piak Nam Yai (PNY) Island, situated in Ranong province, along the Andaman Sea Coast, southwestern Thailand. The MFRC was under the authority of the Department of Marine and Coastal Resources, while the PNY population was under the authority of the Department of National Parks, Wildlife and Plant Conservation, Ministry of Natural Resources and Environment. For 3 years (2020–2022), these two locations were closed to visitors because of the COVID-19 situation. As a result, these *Mfa* populations became less habituated to humans and were roaming freely searching for natural foods. Previously, an extensive survey on the dietary habits of the *Mfa*-PNY island population led to the identification of 47 distinct food items comprised of 43 animal and four plant sources^38^. However, due to the COVID-19 lockdown during our field survey, this assessment focused solely on quantifying the number of distinct food items, without considering their individual proportions. These food items were categorized into natural foods, including marine invertebrates and plants, and anthropogenic foods (Fig. 1). A detailed list of the types of food consumed by these populations is available in Supplementary file 1.

### Nanopore sequencing of bacterial 16S rRNA gene

**Figure 2:**
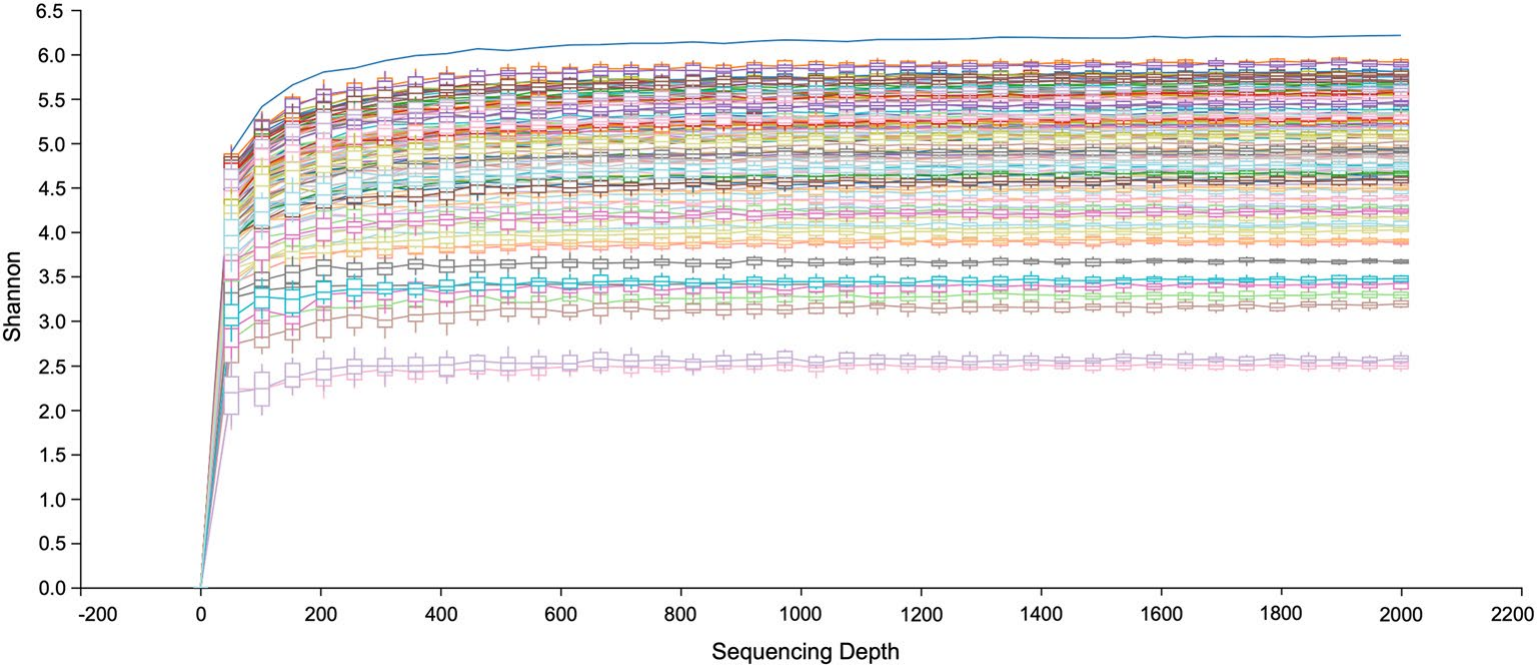
Rarefaction analysis showing that an adequate sequencing depth was obtained for estimating the diversity of all the samples.

The full-length bacterial 16S rRNA gene from 120 fecal samples of long-tailed macaques was successfully sequenced using high throughput nanopore sequencing. In total, 2,444,551 sequencing reads were obtained from 120 samples with an average read per sample of 20,371 (Table 1). The average classified reads were 18,091 per sample. According to the rarefaction analysis, all the samples had sufficient sequencing depth for estimation of the bacterial diversity (Fig. 2).

**Table 1:** Summary of the sequencing and reads classification (mean ± SD) in each population of *Macaca fascicularis fascicularis* (*Mff*) and *M. f. aurea* (*Mfa*) in the two respective habitat types.

Subspecies	Habitat type	Population	Raw read	Retained reads
*Mff*	Mangrove	BTB	21,413 ± 6,693	18,470 ± 5,833
	Island	KPE	19,489 ± 6,298	17,180 ± 5,660
*Mfa*	Mangrove	MFRC	18,402 ± 6,053	16,638 ± 5,552
	Island	PNY	22,179 ± 6,294	20,077 ± 5,745

### Bacterial diversity in the gut microbiome in *Mff* and *Mfa*

**Figure 3:**
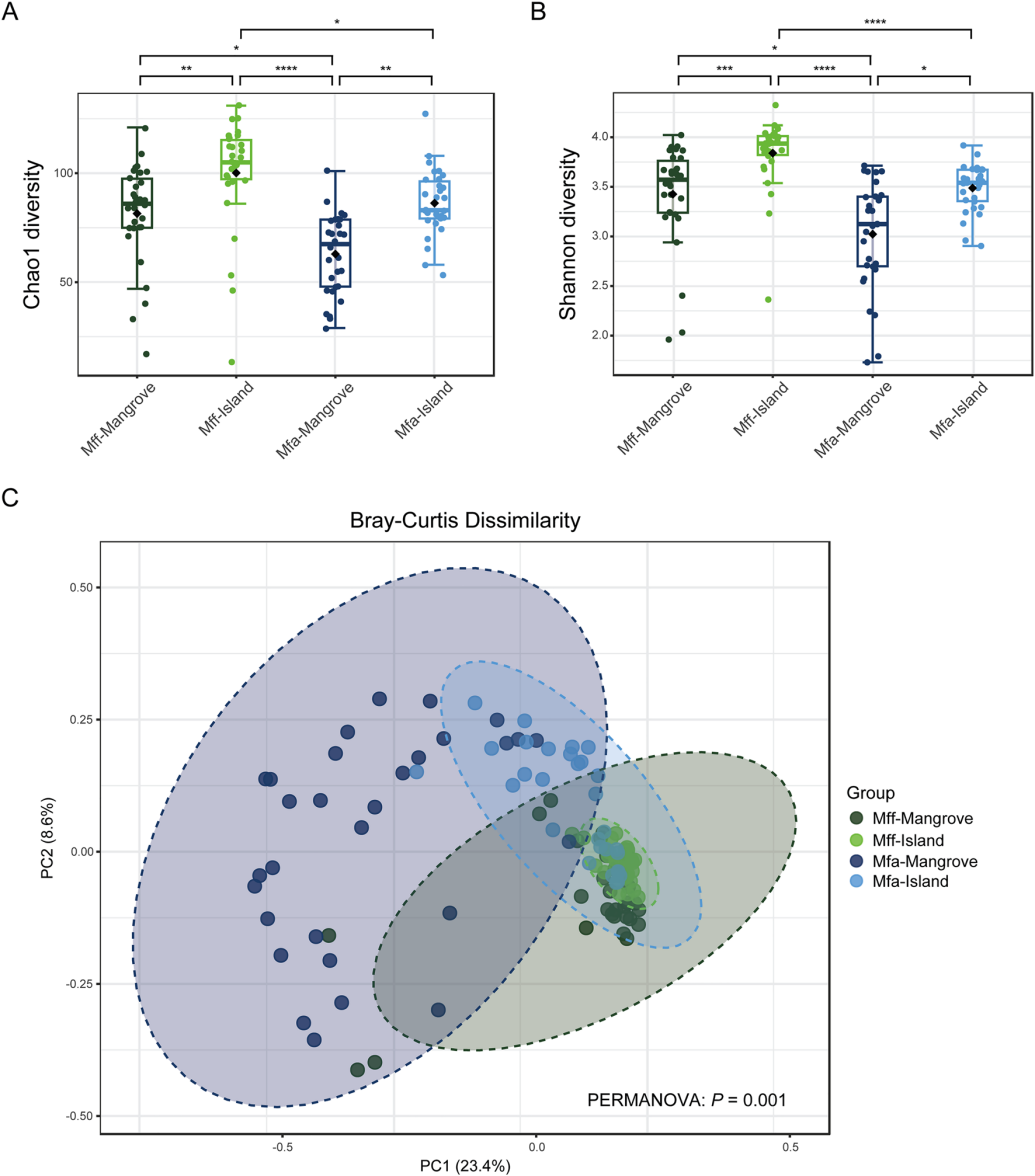
Gut microbiome bacterial diversity in *Mff* and *Mfa* living in mangrove and island habitats. (**a**, **b**) Alpha diversity was compared by the (**a**) Chao1 and (**b**) Shannon indices, while (**c**) Beta diversity was measured by principal coordinate analysis (PCoA) using Bray–Curtis distance. Alpha diversity was statistically tested by Kruskal–Wallis test (*P < 0.05, **P < 0.01, ***P < *0.001,* and **** P < *0.0001*), while PERMANOVA was used for the beta diversity (*P* = *0.001*).

Bacterial alpha diversity (level of diversity within individual samples) comparisons between *Mff* and *Mfa* in the respective mangrove and island populations were evaluated based on the Chao1 index (Fig. 3a), while the richness and evenness of bacterial operational taxonomic units (OTUs) were determined using the Shannon diversity index (Fig. 3b). Statistical comparisons of indices between groups were carried out using a Kruskal–Wallis test, accepting significance at the *P* < *0.05* level.

The Chao1 index and Shannon’s diversity between different habitat types of the *M. fascicularis* subspecies were compared. The Chao1 index of the *Mff*-KPE population on the island had a significantly higher OTU richness (*P* = *0.0021*) than the *Mff*-BTB population in the mangrove forest. Likewise, the Shannon’s diversity was noticeably and significantly higher (*P* = *0.0002*) for the *Mff*-KPE island population than the *Mff*-BTB mangrove population. Similarly, the *Mfa*-PNY population living on the island showed a significantly higher OTU richness (*P* = *0.0021*) and Shannon’s diversity index (*P* = *0.0332*) than the *Mfa*-MFRC mangrove population. Overall, the alpha diversity of *Mff* was significantly higher than that for the *Mfa* populations in both habitat types.

To further examine the differences between the samples, beta diversity (level of diversity or dissimilarity between samples) analyses was performed using the Bray–Curtis cluster analysis index to compare the microbial community compositions between *Mff* and *Mfa* in mangrove and island populations. The beta diversity (Fig. 3c) between *Mff* and *Mfa* in different habitat types (mangrove and island) were significantly different (*P* = *0.001*, permutational multivariate analysis of variance [PERMANOVA]). However, the *Mfa*-MFRC mangrove population had a significant divergence from the other populations.

### Taxonomic composition of the gut microbiota in *Mff* and *Mfa* at different habitats

**Figure 4:**
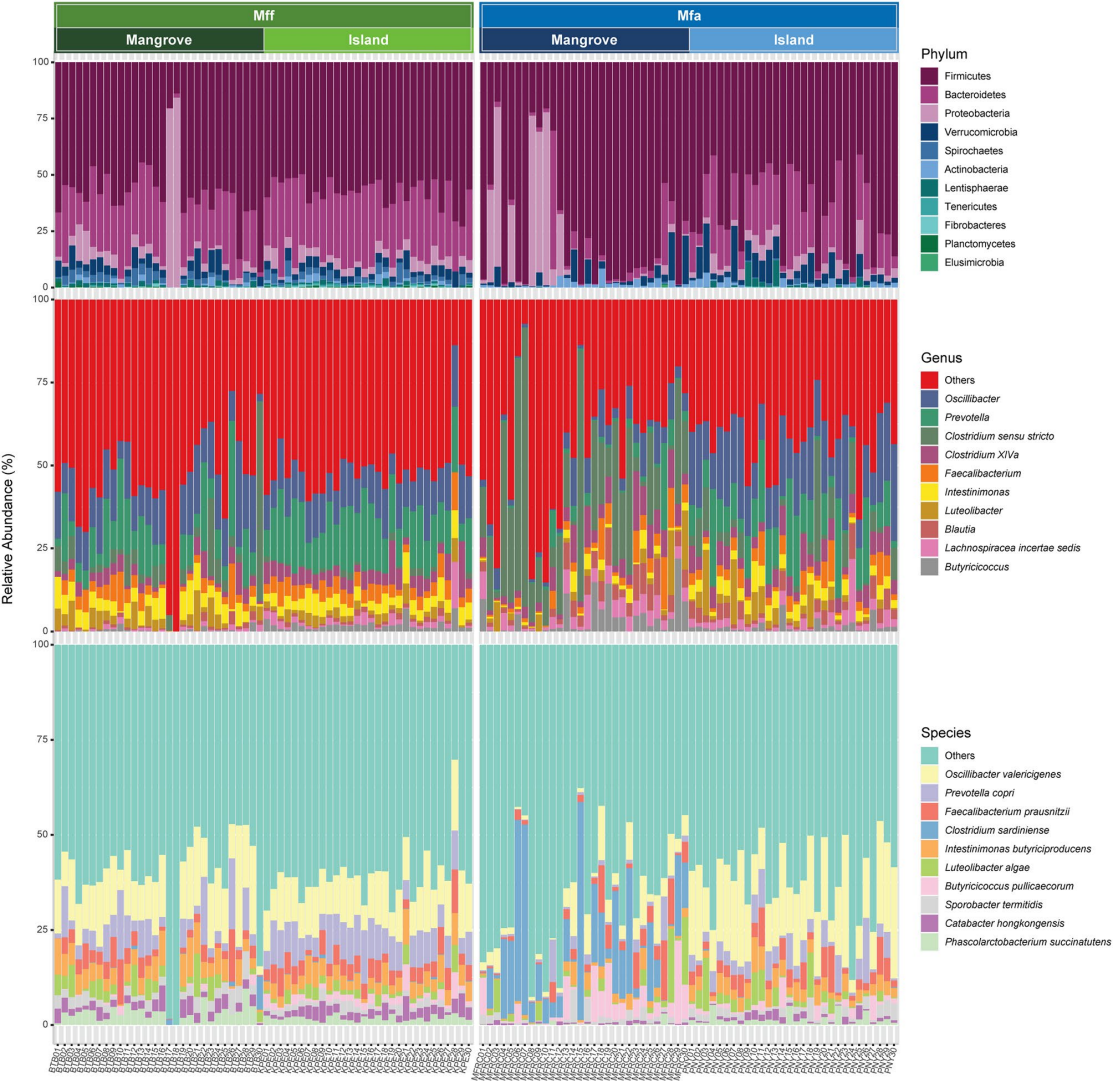
The relative abundance (%; stacked bars) of gut microbiome in *Mff* and *Mfa* living in mangrove and island habitats at the bacterial phyla, genera, and species levels.

Firmicutes was the most dominant bacterial phylum among the mangrove and island *Mff* populations (Fig. 4, upper panel) with a mean ± SD proportion of 57.6 ± 14.6% and 57.3 ± 5.9%, respectively. The Bacteroidetes accounted for 24.0 ± 10.2% and 28.9 ± 6.8% in the *Mff* mangrove and island populations, respectively, making it the second most abundant phylum. However, the relative abundance of Firmicutes and Bacteroidetes was not significantly different between the *Mff* mangrove and island populations (4.7 ± 8.1 and 2.1 ± 1.9 for the mangrove and island populations, respectively; Mann–Whitney U test; *P* < *0.05*). Proteobacteria, which made up 8.7 ± 18.0% and 4.4 ± 2.0% of the bacteriome in the *Mff* mangrove and island populations, respectively, was the third most dominate phylum. Verrucomicrobia, Spirochaetes, Actinobacteria, and Lentisphaerae were among the least abundant phyla in both the *Mff* mangrove and island populations.

Similarly, Firmicutes was the most dominant phylum in the *Mfa* mangrove (74.7 ± 27.2%) and island (64.9 ± 17.9%) populations, while Bacteroidetes was the second most abundant phylum in both *Mfa* populations (5.4 ± 10.7% and 20.6 ± 13.2% for the mangrove and island populations, respectively). In contrast to the *Mff* populations, the relative abundance of Firmicutes to Bacteroidetes ratio in the *Mfa*-PNY island population (8.9 ± 12.4) was significantly lower than the *Mfa*-MFRC-mangrove population (68.3 ± 82.5; Mann–Whitney U test; *P* < 0.001), which was aligned with the higher abundance level of Bacteroidetes in the *Mfa*-PNY island population. The Proteobacteria (14.0 ± 25.8%) and Verrucomicrobia (6.7 ± 6.1%) were the third most abundant phyla in the *Mfa* mangrove and island populations, respectively.

The top 10 most dominant genera in the bacterial communities of both macaque subspecies in the mangrove and island populations were identified and are shown in Fig. 4, middle panel. The most dominant bacterial genus in *Mff* was *Oscillibacter* at 13.3 ± 6.5% and 12.2 ± 3.3% in the mangrove and island populations, respectively. The other predominant bacteria in the bacterial microbiome of *Mff* were *Prevotella*, *Clostridium *sensu stricto, *Clostridium XlVa*, *Faecalibacterium*, and *Intestinimonas*. The proportions of these bacteria varied across samples and were different between the *Mff* mangrove and island populations. *Oscillibacter* was also the most dominant bacterial genus in the fecal microbiome of the *Mfa* island population (17.8 ± 7.6%) and was higher than that in the *Mfa* mangrove population (4.9 ± 4.5%). In contrast, *Clostridium *sensu stricto was the most predominant bacterial genus in the *Mfa* mangrove population (23.1 ± 22.9%) and with a significantly higher abundance (*P* < *0.0001*) than in the *Mfa* island population (4.9 ± 7.4%).

At the bacterial species level, *Oscillibacter valericigenes* was the most dominant species in *Mff* (13.38 ± 6.56% and 12.20 ± 3.36% in the mangrove and island populations, respectively) (Fig. 4, lower panel). The other less dominant bacterial species were *Prevotella copri*, *Intestinimonas butyriciproducens*, and *Faecalibacterium prausnitzii*; however, their abundance varied between populations. In contrast, *Clostridium sardiniense* was the most predominant bacterial species in the *Mfa* mangrove population (14.0 ± 15.5%) with a significantly higher abundance (*P* < 0.00001) than in the *Mfa* island population (0.03 ± 0.1%).

Comparison of the bacterial species between the different macaque subspecies (*Mff* and *Mfa*) in the same habitat types (island or mangrove) was examined by Mann–Whitney U tests (*P* < *0.05*). The results revealed that the Firmicutes and Bacteroidetes were the two most abundant phyla in the *Mfa* and *Mff* island populations; however, the proportion of Firmicutes was not significantly different between them. In contrast, Bacteroidetes were significantly higher in the *Mff* (28.9 ± 6.8%) than in the *Mfa* (20.6 ± 13.2%) island populations. Similarly, the mangrove population of *Mfa* showed a significantly higher abundance of Firmicutes (74.7 ± 27.2%) and a lower abundance of Bacteroidetes (5.4 ± 10.7%) than the *Mff* mangrove population.

The taxonomic bacterial composition at a genera level showed that the *Mfa* island population (17.8 ± 12.2%) had a significantly higher abundance of *Oscillibacter* than the *Mff* island population (12.2 ± 3.3%), while *Clostridium *sensu stricto was significantly higher in the *Mfa* mangrove population (23.1 ± 22.9%) than in the *Mff* mangrove population (5.8 ± 11.1%). Further comparison at the bacterial species level reveled that *Oscillibacter valericigenes* was significantly more abundant in the *Mfa* island population (17.8 ± 7.6%) than in the *Mff* island population (12.2 ± 3.3%), while the *Mfa* mangrove population (14.0 ± 15.5%) had a significantly higher abundance of *Clostridium sardiniense* than the *Mff* mangrove population (0.4 ± 1.6%).

### Differential abundance of gut bacteria between *Mff* and *Mfa* in different habitat types

**Figure 5:**
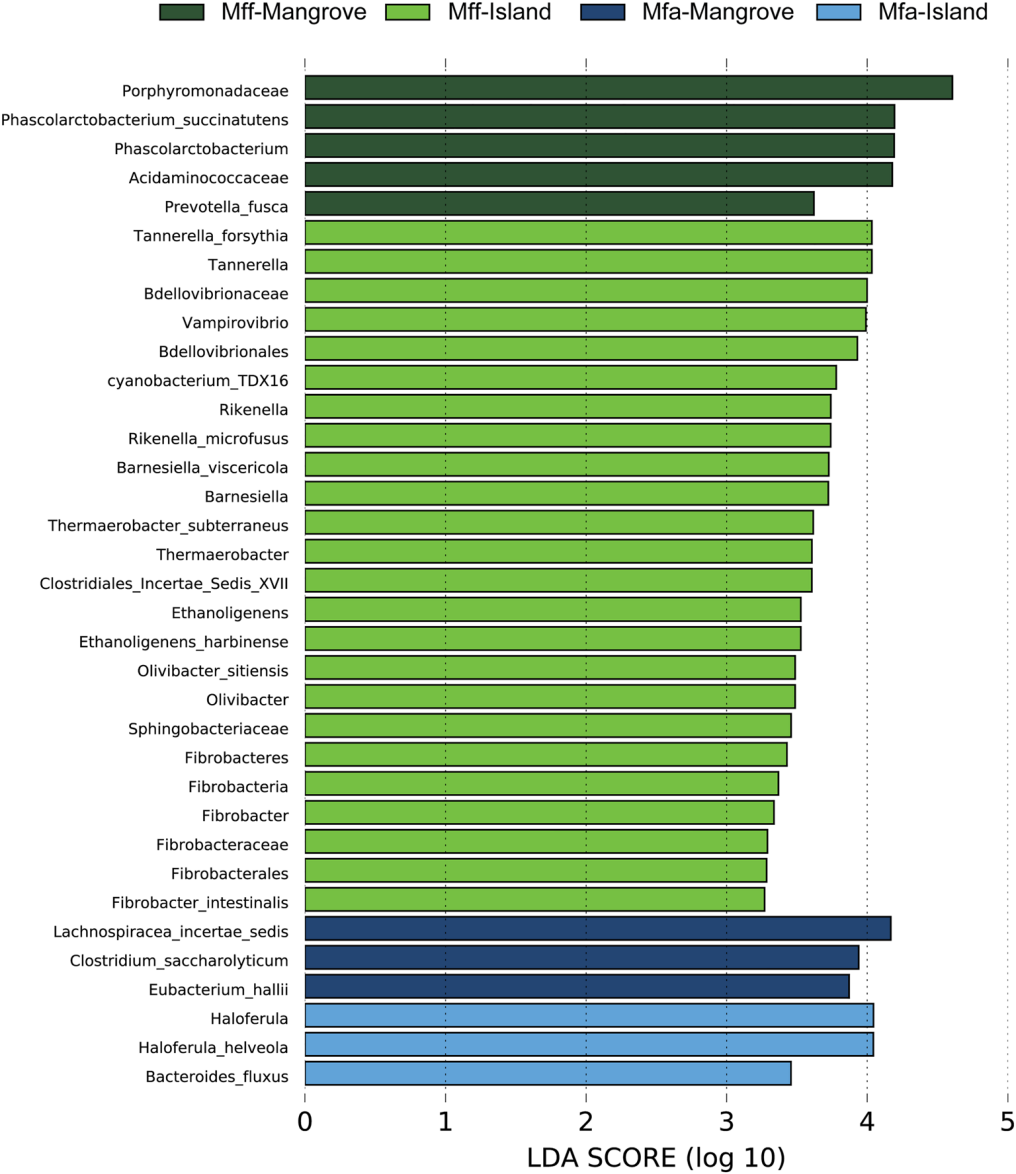
Differential abundance analysis by Linear discriminant analysis Effect Size (LEfSe) of the gut bacterial microbiome of *Mff* and *Mfa* living in a mangrove forest and on the island. The bar plots indicated the differentially abundant bacterial microbiota at different taxonomic ranks. The LDA score shows the effect size and ranking of each differentially abundant taxon (LDA score > 2, *P* < *0.05*).

The taxonomic abundance of the gut bacterial microbiota of *Mff* and *Mfa* living in the mangrove forest and on the island were compared further using LEfSe analysis (LDA score > 2, *P* < *0.05*)^39^, as shown in Fig. 5. Differences in the gut bacterial microbiota between the *Mff* and *Mfa* populations in the different habitat types of mangrove forest and island were identified. *Porphyromonadaceae, Phascolarctobacterium succinatutens, Acidaminococcaceae*, and *Prevotella fusca* were the most enriched taxa in the *Mff*-BTB mangrove population, while the *Mff-*KPE island population had a greater number of significantly enriched taxa, including *Tannerella forsythia, Bdellovibrionaceae, Rikenella microfusus, Barnesiella viscericola, Ethanoligenens harbinense, Olivibacter sitiensis,* and *Fibrobacter intestinalis.* In contrast, the *Mfa*-MFRC mangrove population was enriched in *Lachnospiraceae incertae sedis, Clostridium saccharolyticum,* and *Eubacterium hallii*. Moreover, *Haloferula helveola* and *Bacteroides fluxus* were abundant in the *Mfa*-PNY island population. These results indicate the significant differences in the compositional abundance of gut microbiota between *Mff* and *Mfa* in the mangrove and island populations.

## Discussion

Comparative analysis of the gut bacterial microbiota between *Mff* and *Mfa* living in different habitat types (mangrove forest and island) revealed the potential influence of different environments and diets on their gut bacterial composition. Overall, the gut bacteria’s alpha diversity in *Mff* was significantly higher than in the *Mfa* populations. This difference likely resulted from the increased enrichment of bacterial species in *Mff* populations (BTB and KPE), such as *Oscillibacter valericigenes*, *Prevotella copri*, *Faecalibacterium prausnitzii*, and *Intestinimonas butyriciproducens,* which was primarily influenced by the consumption of anthropogenic foods. A previous study in rhesus macaques (*M. mulatta*) also indicated that the population which consumed anthropogenic foods exhibited a higher microbial richness compared to the wild population that freely foraged for natural foods^40^. Generally, the gut microbial diversity tends to be higher in wild animals compared to captive animals, which is mainly attributed to the complexity of their diet in their natural habitats^41,42^. One possible explanation for the higher bacterial richness in the *Mff* KPE and BTB populations is that, apart from the natural foods in their natural habitats, these *Mff* could access anthropogenic foods regularly. These findings suggest that the gut microbiome’s bacterial composition in *Mff* is primarily influenced by types of food they consume rather than the habitat types they inhabit. Nevertheless, the effect of host (macaque) genetics, which differ between *Mff* and *Mfa*, cannot be ruled out.

The gut microbiota of *Mff* and *Mfa* in this study were mainly composed of two phyla, the Firmicutes and Bacteroidetes, which were most likely similar to that of humans and other NHPs, including other wild and captive Thai *Mff*^34–37,43–46^. Note that the composition of Firmicutes in the *Mfa-*MFRC mangrove population was highest among the four examined populations of long-tailed macaques. Thus, the relative abundance of Firmicutes exhibited variations among different subspecies and habitat types. Specifically, the *Mfa-*MFRC mangrove population showed a higher relative abundance compared to the *Mfa-*PNY island population, and the *Mfa* populations displayed a higher relative abundance compared to the other two *Mff* populations. Firmicute species contained numerous genes encoding enzymes related to energy metabolism, and these bacteria can produce a wide variety of digestive enzymes to decompose various substances, assisting the host in the digestion and absorption of nutrients^47^. According to previous studies, a higher ratio of Firmicutes to Bacteroidetes is associated with a higher absorption of dietary energy^48,49^. Bacteroidetes species helped the host in metabolizing the proteins and carbohydrates in the diet^50,51^. Taken together, it can suggest that the abundance of Firmicutes and the ratio of Firmicutes to Bacteroidetes are related to the genetic characteristics (leading to a different subspecies of *Mff* and *Mfa*), habitat type (mangrove forests or island), and anthropogenic foods (only in *Mff* populations). The higher abundance of Firmicutes to Bacteroidetes may partially be related to the consumption of the high-energy mollusk foods that were observed to be heavily consumed in the *Mfa* populations in this study. These *Mfa* populations were observed to primarily rely on the natural food sources available in their respective habitats subject to their specific foraging techniques to acquire these foods. In addition, the abundance of bacteria belonging to the phylum Proteobacteria in the *Mfa*-MFRC mangrove population was significantly higher than in the *Mfa*-PNY island population, which could reflect the effects of the habitat type and food items.

During fecal specimen collections, we discovered that the *Mff*-KPE populations, especially adults, sporadically used percussive stone tools for opening oysters. Thus, the higher bacterial species richness observed in the *Mff*-KPE population can be attributed to their consumption of anthropogenic foods and their stone-tool use behavior, which allows them to access more food items requiring foraging techniques. This indicates that while diet plays a significant role in bacterial diversity, stone-tool use behavior also contributes to the bacterial diversity. However, due to the short-time stay and lack of individual animal identification and stone-tool use in this study, we were unable to collect data on the proportion of food types consumed by the monkeys on a daily basis. Besides, the data on stone-tool use by each population was obtained from previous studies^24,27,38^, and were also confirmed during the field observations. This limitation hinders our ability to analyze the microbiome composition at an individual level based on the proportion of food consumption. To address this limitation in future research, it would be beneficial to identify each animal individually, collect data on the proportion of their daily food consumption with or without stone-tool use, and then analyze the microbiome composition at the individual level. Thus, collecting data on the proportion of food items acquired through stone-tool use and without the use of stone-tools for each individual animal would allow for a more detailed analysis of the relationship between stone-tool use, dietary habits, and the gut microbial profiles. Such an individual-level analysis would provide valuable insights into how specific dietary behaviors shape the gut microbiota within each population of *M. fascicularis*, contributing to a deeper understanding of the factors driving gut microbiome variation in these macaque populations.

At the genus level, our results indicated that the microbiome of long-tailed macaque populations was enriched with *Prevotella*, which is one of the most predominant genera in the human microbiome. In line with these findings, a previous study also reported that the macaque microbiome exhibited a higher abundance of *Prevotella* than the human microbiome^52^. The predominance of *Prevotella* was associated with a diet high in carbohydrate and fiber from plant sources^53^. Similarly, western lowland gorillas (*Gorilla gorilla gorilla*) that consumed a high number of fruits had a high relative abundance of Prevotellaceae^54^. These findings suggest that populations with a higher abundance of *Prevotella* possess the capacity to effectively break down and utilize the natural plant-based diet.

At the bacterial species level, *Oscillibacter valericigenes* was the most dominant species in the *Mfa* and *Mff* populations with the exception in the *Mfa*-MFRC mangrove population. *Oscillibacter valericigenes* is a representative bacterium in the Oscillibacter group that can produce valerate^55^, a short-chain fatty acid that can replace butyrate as an energy source for colonocytes. This bacterium’s abundance showed its potential relevance to the macaque’s health. These results are also consistent with a previous study reporting a significant abundance of *O. valericigenes* in healthy humans^56^. Similarly, *Faecalibacterium prausnitzii* was present in all four populations of long-tailed macaques examined in this study, which is supported by previous studies that *F. prausnitzii* was the dominant butyrate producer of *Clostridium cluster IV*, the most common bacteria in the microbiome of humans, and which exhibited anti-inflammatory effects^57^ and enhanced the gut barrier functions^58^. The depletion of *F. prausnitzii* is associated with Chron’s disease^59^. Note that the microbiome of the *Mfa*-MFRC mangrove population was enriched with *Clostridium sardiniense* and less diversified. These findings are significant for the health of long-tailed macaques because a reduced diversity in the gut microbiota results in fewer microbial metabolic pathways interacting with food items and providing fewer nutritional benefits to the hosts. Similarly, in other mammalian species, a low gut microbial diversity has also been associated with heightened vulnerability to opportunistic pathogens^60^. *Clostridium sardiniense* is a glycolytic cluster I species that uses anaerobic carbohydrate fermentation to produce butyrate^61^. This species can also promote a more severe infection of *Clostridioides difficile* in mice by modulating the virulence, growth, and colonization of the pathogen^62^. Also, the reduced Chao 1 and Shannon alpha diversity of the microbiome in the *Mfa-*MFRC mangrove population could potentially be attributed to the higher abundance of *C. sardiniense*. It is essential to highlight that the *Mfa-*MFRC mangrove population in this study are wild animals and are not habituated to human presence. Due to COVID-19 restrictions, human activities were limited during the field observations of *Mfa-*MFRC mangrove population. As a result, these animals predominantly relied on natural food sources, leading to a less diverse range of microbial species compared to the *Mff* populations, which had access to both natural and anthropogenic foods.

The LEfSe-based differential species abundance analysis of the *Mff*-BTB mangrove population revealed that Porphyromonadaceae and *Phascolarctobacterium succinatutens* were the most enriched taxa. The Porphyromonadaceae have a potential role as adiposity modulators by producing two short-chain fatty acids: acetate and propionate^63,64^. *Phascolarctobacterium succinatutens* is known for its utilization of succinate and has been previously identified in the gut of healthy humans^64^. These results suggest that the *Mff*-BTB mangrove population have a specific diet that promotes the growth and proliferation of Porphyromonadaceae and *P. succinatutens*. These bacteria are known to thrive on certain dietary components, such as complex carbohydrates and fibers, which are abundant in the macaques’ food sources in the mangrove habitat.

*Tannerella forsythia*, a well-known oral human pathogen^65^, was found to be more abundant in the *Mff*-KPE island population, as indicated by the LEfSe analysis*.* Periodontitis in humans is strongly associated with the presence of *T. forsythia* and this species has a significant role in the pathogenicity of the microbiota in subgingival plaques^66^. In the short-time observations during fecal specimen collection and our previous observations before the COVID-19 episode, the *Mff*-KPE island population was seen to be heavily provided with fresh and leftover foods by humans compared to the other three macaque populations. Thus, it is possible that their diet, which includes anthropogenic food, might have contributed to their higher abundance of *T. forsythia* in the gut microbiota.

Following the LEfSe analysis, the *Mfa-*PNY island population, which did not receive anthropogenic foods, showed an enrichment of *Haloferula helveola* and *Bacteroides fluxus*. *Haloferula helveola* is commonly associated with marine environments^67^, and is not known to inhabit the human gut in any marked abundance according to the data from the U.S. NIH Human Microbiome Project^68^ and the search engine of EZBioCloud^69^. This is in accord with a previous report that indicated that marine invertebrates were the main food source of the *Mfa-*PNY island population^38^. *Bacteroides fluxus* has been isolated from the feces of healthy human individuals^70^. Nevertheless, one case of its presence in an abdominal infection has been reported^71^. Overall, the higher abundance of these bacterial species in the *Mfa-*PNY island population can be attributed to their specific diet, which includes marine-based foods, and their adaptation to a distinct island habitat, which likely influenced the composition of their gut microbiome.

According to the LEfSe analysis, the *Mfa*-MFRC mangrove population was enriched with bacterial species from the family Lachnospiraceae. These bacterial species are known to degrade complex polysaccharides, producing butyrate that can be utilized for energy^72^. This finding aligns with the dietary habits of herbivores, which are known to have a higher abundance of Lachnospiraceae compared to omnivores^73^. The results may reflect the plant-based dietary sources available to the *Mfa*-MFRC mangrove population in their habitat.

In conclusion, this is the first report to compare the gut microbiomes of different subspecies of *M. fascicularis* (*Mff* and *Mfa*) living in two different habitat types (mangrove forest and island). The results revealed a significant difference in the gut microbiome associated with the different genetic background of the animals (between the two subspecies of *M. fascicularis*) and their diverse dietary habits (comparing between mangrove forest and island habitats, as well as anthropogenic foods). The latter factor could be associated with the use of stone-tools in foraging for foods. It was previously reported that the *Mfa*-PNY island population used percussive stone tools daily^24,32,38^, while the *Mfa*-MFRC mangrove population performed only food-pounding behaviors^30^. The food-pounding behavior is when the animals used the food (i.e., shell) to pound the food or to pound the stone, while the stone-tool use behavior is using the stone to pound the food, as seen in the *Mfa*-PNY macaques^30^. Furthermore, the study offered intriguing insights into the potential influences of stone-tool use and anthropogenic foods on the macaque’s health, as evidenced through their gut microbiome. The higher gut bacterial diversity observed in the *Mff* populations, especially in *Mff*-KPE island population with access to anthropogenic foods and stone-tool use behavior, suggested that both diet and stone-tool use play significant roles in shaping the gut microbiome. In contrast, the reduced diversity in the *Mfa*-MFRC mangrove population that relies solely on natural food sources may reflect limitations in accessing a diverse range of microbial species. However, to comprehensively elucidate the influences of stone-tool use and diet acquisition on macaque health through the microbiome, further research is needed to investigate the individual-level relationship between stone-tool use, dietary habits, and gut microbial profiles. This is the next question for us to explore further.

## Methods

### Permit and ethical note

The permits for research and sample collection in the four populations of free-ranging long-tailed macaques sampled in this study in Thailand were approved by the Department of National Parks, Wildlife, and Plant Conservation of Thailand. The Institutional Animal Care and Use Committee (IACUC) of the National Primate Research Center of Thailand-Chulalongkorn University approved the study’s experimental protocols (Protocol Review no. 2075007). The research adhered to the American Society of Primatologists (ASP) Principles for the Ethical Treatment of Non-Human Primates. All methods were performed in accordance with the relevant guidelines and regulations.

### Study sites and consumed food items

Two subspecies of free-ranging *Macaca fascicularis* (*Mff* and *Mfa*) at two habitat types (island and mangrove forest), giving a total of four populations, in Thailand were selected for this study (Table 2). The subspecies were identified based on their geographical distribution and morphological characteristics^23,27,30^. The information regarding the food consumed by the monkeys was gathered through direct observation of foraging animals and their consumed foods, or by observing the remaining food item(s) after the animals had finished eating. Food items were identified and photographed using a Nikon COOLPIX W300 (Nikon, Japan).

**Table 2:** Subspecies, code, location, geographical coordinate, habitat types, and date of specimen collection of the wild *Mff* and *Mfa* populations in this study.

Subspecies	Code	Location	GPS (N, E)	Habitat	Specimen collection
*Mff*	KPE	Koh Ped, Chonburi	12°45′, 100°50′	Island	12–16 July, 2022
	BTB	Bang Ta Boon, Phetchaburi	13°15′, 99°56′	Mangrove	17–21 July, 2022
*Mfa*	MFRC	Mangrove Forest Research Center, Ranong	9°52′, 98°36′	Mangrove	26–30 July, 2022
	PNY	Piak Nam Yai, Ranong	9°35′, 98°28′	Island	31 July to 6 Aug, 2022

### Fecal specimen collection

A total of 120 freshly defecated specimens (n = 30 for each population) were non-invasively collected using the fecal swab method in their natural habitats. In each location, the survey was conducted over at least five consecutive days, at 7:00–16:00 h (see Table 2). To avoid contamination with the soil microbiome, the fecal samples were collected from the inner part using cotton swabs (Citoswab, China). Samples were preserved in 2 mL of DNA/RNA shield (Zymo Research, USA) for viral inactivation and nucleic acid stabilization. To avoid double collection, the physical characteristics (i.e., color, texture, and shape) of each fecal specimen were recorded.

### DNA extraction

DNA was extracted using ZymoBIOMICS™ DNA Miniprep kit (Zymo Research, USA). Briefly, 750 µL of fecal suspension were lysed in a ZR BashingBead™ lysis tube using TissueLyser LT (Qiagen, Germany) at 50 Hz for 3 min. The cell lysate was then extracted following the manufacturer’s instruction. The concentration of DNA was determined using A_260_/_280 nm_ by NanoPhotometer® C40 (Implen, Germany).

### PCR amplification and sequencing on MinION™

The full length bacterial 16S small subunit ribosomal RNA (16S rRNA) gene, *ca*. 1,500-bp size, was amplified based on PCR with the specific primers; 16S-V1F 5′-TTTCTGTTGGTGCTGATATTGCAGRGTTYGATYMTGGCTCAG-3′ and 16S-V9R 5′-ACTTGCCTGTCGCTCTATCTTCCGGYTACCTTGTTACGACTT-3′^74^. The 10 µL PCR reaction mixture consisted of 5 µL of 2 × UltraHiFi mix (Tiangen, China), 2 µL of PCR Enhancer (Tiangen, China), 0.25 µM each of forward and reverse primers, 1.5 µL of ddH_2_O, and 1 µL of the nucleic acid template. The PCR was thermal cycled at 94 °C for 2 min, followed by 25 cycles of 98 °C for 10 s, 60 °C for 30 s, and 68 °C for 45 s, and then a final 68 °C for 5 min. The amplicons were barcoded by a five-cycle PCR using the barcode primers based on the PCR Barcoding Expansion 1–96 kit (EXP-PBC096; Oxford Nanopore Technologies, UK). The barcoded libraries were enriched using a QIAquick® PCR Purification kit (QIAGEN, Germany) following the manufacturer’s instructions. The enriched libraries were quantified by Quant-iT™ dsDNA HS Assay kit using Qubit 4 fluorometer (Invitrogen, USA), and then equimolarly pooled for multiplexing. The pooled library was enriched using 0.5 × Agencourt AMPure XP beads (Beckman Coulter, USA). Afterwards, the library was subjected to end repair and adaptor ligation steps using Ligation Sequencing Kit (SQK-LSK114). Finally, the library was loaded onto the R10.4.1 flow cell and sequenced on a MinION™ Mk1C sequencer (Oxford Nanopore Technologies, UK).

### Data analysis

The FASTQ files were generated from the FAST5 data based on a super-accuracy model with a minimum acceptability quality score (Q > 10) using the Guppy basecaller software v6.0.7 (Oxford Nanopore Technologies, UK)^75^, while MinIONQC was used for the evaluation of the quality of the reads^76^. Porechop v0.2.4 was used for adaptor-trimming and demultiplexing of FASTQ sequences^77^. NanoCLUST was used for clustering, polishing, and taxonomically classifying the filtered reads, based on the size of the sequences for the V1–V9 region of 16S rRNA gene sequences from the Ribosomal Database Project (RDP) database^78,79^. The files were converted into QIIME (Quantitative insight into microbial ecology) format, and the QIIME2 toolkit v2021.2 was used for calculation of the alpha diversity using Chao1 and Shannon indices, and the beta diversity by Bray–Curtis cluster analysis^80^. The MicrobiomeAnalyst was used for the visualization of normalized data^81^. Finally, the Galaxy server was used for the differential abundance analysis of gut microbiota using linear discriminant analysis Effect Size (LEfSe) with *P* < *0.05* and a linear discriminant analysis (LDA) score > 2^39^.

### Supplementary Information

Supplementary Information.

## Data Availability

The datasets generated from the next-generation sequencing in this study are available in the NCBI Sequence Read Archive (SRA) repository, Bioproject ID: PRJNA926923 or https://www.ncbi.nlm.nih.gov/bioproject/PRJNA926923
