# Supplementary material for: Comparative analysis of gut microbiota between common (Macaca fascicularis fascicularis) and Burmese (M. f. aurea) long-tailed macaques in different habitats
Source: Sci Rep. 2023 Sep 11;13:14950. doi: 10.1038/s41598-023-42220-z (PMC10495367; doi:10.1038/s41598-023-42220-z)
Supplement: Supplementary file 1 — Supplementary Information. [file 41598_2023_42220_MOESM1_ESM.docx]

**Comparative analysis of gut microbiota between common (*Macaca fascicularis fascicularis*) and Burmese (*M. f. aurea*) long-tailed macaques in different habitats**

Raza Muhammad^1^, Pavit Klomkliew^2^, Prangwalai Chanchaem^2^, Vorthon Sawaswong^2^, Titiporn Kaikaew^1^, Sunchai Payungporn^2,3,*^, Suchinda Malaivijitnond^1,4,*^

^1^Department of Biology, Faculty of Science, Chulalongkorn University, Bangkok 10330, Thailand.

^2^Center of Excellence in Systems Microbiology, Faculty of Medicine, Chulalongkorn University, Bangkok 10330, Thailand.

^3^Department of Biochemistry, Faculty of Medicine, Chulalongkorn University, Bangkok 10330, Thailand.

^4^National Primate Research Center of Thailand, Chulalongkorn University, Saraburi 18110, Thailand.

*Correspondence email: [suchinda.m@chula.ac.th](mailto:suchinda.m@chula.ac.th); [sp.medbiochemcu@gmail.com](mailto:sp.medbiochemcu@gmail.com)

**Supplementary Table S1** A list of food types consumed by common long-tailed macaques (*Mff*) at BTB mangrove forest.

| **Natural foods** | | | | | | | |
| --- | --- | --- | --- | --- | --- | --- | --- |
| **No** | **Food group** | **Kingdom** | **Phylum** | **Class** | **Genera** | **Specie** | **Vernacular names** |
|  | Marine invertebrate | Animalia | Mollusca | Bivalvia | *Saccostrea* | *cucullata* | Rock oyster |
|  | Plant | Plantae | Tracheophyta | Magnoliopsida | *Avicennia* | *alba* | Leaf |
|  | Plant | Plantae | Tracheophyta | Magnoliopsida | *Avicennia* | *alba* | Fruit |
|  | Plant | Plantae | Spermatophyta | Dicotyledonae | *Amaranthus* | *viridis* | Slender amaranth |
|  | Plant | Plantae | Spermatophyta | Dicotyledonae | *Achyranthes* | *aspera* | Leaf |
| **Anthropogenic foods** | | | | | | | |
|  | Banana | | | | | | |
|  | Guava | | | | | | |
|  | Watermelon | | | | | | |

**Supplementary Table S2** A list of food types consumed by common long-tailed macaques (*Mff*) at KPE Island.

| **Natural foods** | | | | | | | |
| --- | --- | --- | --- | --- | --- | --- | --- |
| **No** | **Food group** | **Kingdom** | **Phylum** | **Class** | **Genera** | **Specie** | **Vernacular names** |
|  | Marine invertebrate | Animalia | Mollusca | Bivalvia | *Saccostrea* | forskali | Rock oyster |
|  | Marine invertebrate | Animalia | Mollusca | Bivalvia | *Pinna* | *bicolor* | Bicolored pen shell |
|  | Marine invertebrate | Animalia | Mollusca | Bivalvia | *Anadara* | *granosa* | Blood clam |
|  | Marine invertebrate | Animalia | Mollusca | Bivalvia | *Anadara* | *inaequivalvis* | Hairy cockle |
|  | Marine invertebrate | Animalia | Mollusca | Bivalvia | *Gafrarium* | *-* |  |
|  | Marine invertebrate | Animalia | Arthropoda | Malacostraca |  | *-* | Crab |
|  | Marine invertebrate | Animalia | Chordata | *-* | *-* | *-* | Fish |
|  | Plant | Plantae | Spermatophyta | Monocotyledoneae | *Zea* | *mays* | Corn |
|  | Plant | Plantae | Spermatophyta | Dicotyledonae | *Coccinia* | *grandis* | Scarlet gourd |
|  | Plant | Plantae | Spermatophyta | Dicotyledonae | *Colubrina* | *asiatica* | Latherleaf |
|  | Plant | Plantae | Spermatophyta | Dicotyledonae | *Sida* | *acuta* | Sida |
|  | Plant | Plantae | *-* | *-* | *-* | *-* | Leaf |
| **Anthropogenic foods** | | | | | | | |
|  | Watermelon | | | | | | |
|  | Banana | | | | | | |
|  | Pineapple | | | | | | |

**Supplementary Table S3** A list of food types consumed by Burmese long-tailed macaques (*Mfa*) at MFRC mangrove forest.

| **Natural foods** | | | | | | | | |
| --- | --- | --- | --- | --- | --- | --- | --- | --- |
| **No** | **Food group** | **Kingdom** | **Phylum** | **Class** | **Genera** | **Specie** | **Vernacular names** |  |
|  | Marine invertebrate | Animalia | Mollusca | Bivalvia | *Saccostrea* | *cucullata* | Rock oyster |  |
|  | Marine invertebrate | Animalia | Mollusca | Bivalvia | *Geloina* | *-* | - |  |
|  | Marine invertebrate | Animalia | Arthropoda | Malacostraca | *Grapsus* | *albolineatus* | Shore crab |  |
|  | Marine invertebrate | Animalia | Arthropoda | Malacostraca | *-* | *-* | Crab |  |
|  | Plant | Plantae | Spermatophyta | Dicotyledonae | *Rhizophora* | *mucronata* | loop-root mangrove |  |
|  | Plant | Plantae | Spermatophyta | Dicotyledonae | *Excoecaria* | *agallocha* | Milky mangrove |  |
|  | Plant | Plantae | Tracheophyta | Magnoliopsida | *Xylocarpus* | *moluccensis* | Nyireh batu |  |
|  | Plant | Plantae | Tracheophyta | Magnoliopsida | *Xylocarpus* | *granatum* | Cannonball mangrove |  |
|  | Plant | Plantae | Tracheophyta | Magnoliopsida | *Ceriops* | *tagal* | Spurred mangrove |  |

**Supplementary Table S4** A list of food types consumed by Burmese long-tailed macaques (*Mfa*) at PNY Island.

| **Natural foods** | | | | | | | | |
| --- | --- | --- | --- | --- | --- | --- | --- | --- |
| **No** | **Food group** | **Kingdom** | **Phylum** | **Class** | **Genera** | **Specie** | **Vernacular names** |  |
|  | Marine invertebrate | Animalia | Mollusca | Bivalvia | *Saccostrea* | *cucullata* | Rock oyster |  |
|  | Marine invertebrate | Animalia | Mollusca | Bivalvia | *Barbatia* | *-* | - |  |
|  | Marine invertebrate | Animalia | Mollusca | Bivalvia | *Ruditapes* | *-* | - |  |
|  | Marine invertebrate | Animalia | Mollusca | Bivalvia | *Gafrarium* | *divaricatum* | forked Venus |  |
|  | Marine invertebrate | Animalia | Mollusca | Bivalvia | *Asaphis* | *violascens* | Pacific asaphis |  |
|  | Marine invertebrate | Animalia | Mollusca | Gastropoda | *Nerita* | *chamaeleon* | Chameleon nerite |  |
|  | Marine invertebrate | Animalia | Mollusca | Gastropoda | *Pugilina* | *cochlidium* | Crown conch |  |
|  | Marine invertebrate | Animalia | Mollusca | Gastropoda | *Laevistrombus* | *canarium* | Dog conch |  |
|  | Marine invertebrate | Animalia | Arthropoda | Malacostraca | *Thalamita* | *-* | Swimming crab |  |
|  | Plant | Plantae | Magnoliophyta | Magnoliopsida | *Terminalia* | *catappa* | Sea almond fruiting |  |
|  | Plant | Plantae | Magnoliophyta | Liliopsida | *Pandanus* | *tectorius* | Seashore pandan |  |
|  | Plant | Plantae | Spermatophyta | Monocotyledoneae | *Cocos* | *nucifera* | Coconut |  |
|  | Plant | Plantae | Spermatophyta | Dicotyledonae | *Parkia* | *speciosa* | Bitter bean |  |
|  | Plant | Plantae | Spermatophyta | Dicotyledonae | *Rhizophora* | *mucronata* | loop-root mangrove |  |
|  | Plant | Plantae | - | - | *-* | *-* | Fiber |  |
